# Feasibility of Low-Dose and Low-Contrast Media Volume Approach in Computed Tomography Cardiovascular Imaging Reconstructed with Model-Based Algorithm

**DOI:** 10.3390/tomography10020023

**Published:** 2024-02-16

**Authors:** Davide Ippolito, Marco Porta, Cesare Maino, Luca Riva, Maria Ragusi, Teresa Giandola, Paolo Niccolò Franco, Cecilia Cangiotti, Davide Gandola, Andrea De Vito, Cammillo Talei Franzesi, Rocco Corso

**Affiliations:** 1 Departement of Medicine and Surgery, University of Milano-Bicocca, Piazza OMS 1, 20100 Milano, Italy; davide.atena@tiscalinet.it; 2 Department of Diagnostic Radiology, Fondazione IRCCS Fondazione San Gerardo dei Tintori, Via Pergolesi 33, 20900 Monza, Italy; porta.marco90@gmail.com (M.P.); luca.riva@asst-lecco.it (L.R.); maria.ragusi@gmail.com (M.R.); teresagiandola1990@gmail.com (T.G.); francopaoloniccolo@gmail.com (P.N.F.); cecilia.cangiotti@irccs-sangerardo.it (C.C.); gandolad@gmail.com (D.G.); andrea.devito@irccs-sangerardo.it (A.D.V.); ctfdoc@gmail.com (C.T.F.); rocco.corso@irccs-sangerardo.it (R.C.)

**Keywords:** radiation exposure, algorithms, tomography, X-ray computed, model-based iterative reconstruction, computed tomography angiography, transcatheter aortic valve implantation

## Abstract

**Aim**: To evaluate the dose reduction and image quality of low-dose, low-contrast media volume in computed tomography (CT) examinations reconstructed with the model-based iterative reconstruction (MBIR) algorithm in comparison with the hybrid iterative (HIR) one. **Methods:** We prospectively enrolled a total of 401 patients referred for cardiovascular CT, evaluated with a 256-MDCT scan with a low kVp (80 kVp) reconstructed with an MBIR (study group) or a standard HIR protocol (100 kVp—control group) after injection of a fixed dose of contrast medium volume. Vessel contrast enhancement and image noise were measured by placing the region of interest (ROI) in the left ventricle, ascending aorta; left, right and circumflex coronary arteries; main, right and left pulmonary arteries; aortic arch; and abdominal aorta. The signal-to-noise ratio (SNR) and contrast-to-noise ratio (CNR) were computed. Subjective image quality obtained by consensus was assessed by using a 4-point Likert scale. Radiation dose exposure was recorded. **Results:** HU values of the proximal tract of all coronary arteries; main, right and left pulmonary arteries; and of the aorta were significantly higher in the study group than in the control group (*p* < 0.05), while the noise was significantly lower (*p* < 0.05). SNR and CNR values in all anatomic districts were significantly higher in the study group (*p* < 0.05). MBIR subjective image quality was significantly higher than HIR in CCTA and CTPA protocols (*p* < 0.05). Radiation dose was significantly lower in the study group (*p* < 0.05). **Conclusions:** The MBIR algorithm combined with low-kVp can help reduce radiation dose exposure, reduce noise, and increase objective and subjective image quality.

## 1. Introduction

Computed tomography angiography (CTA) is thought to be an effective method for evaluating aortic, pulmonary, and coronary artery disease. It is specifically advised to use coronary CTA (CCTA) as the first test when detecting coronary artery disease (CAD) [1,2]. When a patient is suspected of having a pulmonary embolism (PE), CT pulmonary angiography (CTPA) is thought to be the primary imaging method. 

Finally, CTA can help evaluate aortic anatomy and pathology, both in acute settings, such as in the case of aneurysm rupture or dissection, and in outpatients, to plan transcatheter aortic valve replacement (TAVR), as reported by the most important international consensus statements and guidelines [3,4,5].

CT has become a fundamental imaging technique and, consequently, radiation dose exposure and contrast media (CM) administration have rapidly increased during the last few years, as reported by the European Society of Cardiology guidelines published in 2019 [6]. In clinical practice, it is important to balance radiation dose exposure and image quality: CT scans should be technically designed to keep the radiation dose as low as possible and to provide the required diagnostic information, according to the ALARA principle [7]. To minimize radiation dose exposure, CT protocols should be set by adjusting technical acquisition parameters, including the application of different noise filters [8], modifying pitch values [9], tube voltage (kVp), and current (mAs) [10,11]. All the proposed methods are linked to image acquisition, with the exception of noise filters and reconstruction algorithms. The latter help to increase image quality after the acquisition process is completed. For this reason, hybrid iterative reconstruction (HIR) algorithms have been introduced to improve image quality, especially in low-dose protocols, allowing for a dose reduction of about 50% [10,11]. Furthermore, the newer model-based iterative reconstruction (MBIR) algorithms were introduced to increase the spatial resolution and to reduce image noise [12]. The hybrid iterative process is based on iterations in the sinogram domain, one back projection, and more iterations in the image domain. The one back projection is not significantly computationally demanding, and is also less capable in terms of noise and artifact reduction; consequently, noise is penalized and edges are preserved. On the other hand, the MBIR algorithm uses multiple iterations of forward and back projections between the sinogram domain and image domain to optimize image quality. Moreover, models of acquisition process, noise statistics, and system geometry reconstruct the projections as accurately as possible in order to obtain a greater reduction in noise and artifacts than other reconstruction algorithms, thus resulting in high-quality images even in lower-dose protocols [13,14].

To gain an almost-perfect balance between radiation dose exposure and the risk of renal failure, it is fundamental to deal with the contrast media volume, especially in patients with different comorbidities, as suggested by the ESUR guidelines 10.0 [15].

Finally, the setting of low-kVp protocols allows for further reduction in the CM volume due to greater photoelectric effect and decreased Compton scattering [16,17,18,19,20].

On these bases, this study aims to determine the usefulness of CT low-kVp protocols with fixed CM volumes in cardiovascular imaging by comparing the application of MBIR and HIR algorithms, and their effects on the radiation dose exposure.

## 2. Materials and Methods

### 2.1. Study Population

This study was performed in a single university center and tertiary referral hospital and was approved by the Ethical Committee of Institutional Review Board (105-RDX-FI-0731); written consent was obtained from each participant.

From November 2016 to December 2019, all consecutive patients who underwent CCTA, CTPA, and pre-TAVR CTA were prospectively enrolled and randomly allocated (ratio 1:1) into study and control groups. Before being divided into the two groups, we applied inclusion and exclusion criteria, as reported below.

Inclusion criteria for CCTA were as follows: (1) detection of CAD, (2) coronary assessment before surgery, (3) prior stress-imaging procedures with discordant electrocardiographic exercise and imaging results or equivocal stress-imaging results, (4) risk assessment post-revascularization, (5) evaluation of cardiac anatomy, (6) diagnosis of in-stent restenosis, (7) evaluation of coronary bypass graft patency.

Inclusion criteria for CTPA and pre-TAVR CTA were suspected pulmonary embolism, both acute and chronic, and preoperative anatomy assessment, respectively.

Overall exclusion criteria were as follows: (1) age < 18 years, (2) severe renal failure (eGFR < 30 mL/min/1.73 m^2^), (3) contraindications for iodinated contrast materials (e.g., previous allergic reaction).

Specific exclusion criteria for CCTA were as follows: (1) unstable angina or previous coronary interventions, (2) BMI value > 30 kg/m^2^, (3) heart rate > 65 bpm with contraindications to the use of β-blocker or arrhythmia. Specific exclusion criteria for pre-TAVR CTA were as follows: (1) BMI value > 35 kg/m^2^, and (2) heart rate > 85 bpm.

### 2.2. CT Protocols

All patients were evaluated with a 256-MDCT scan examination (iCT Elite, Philips Medical Systems, Best, The Netherlands) with a low-kVp (80 kVp) protocol in the study group and a standard (100 kVp) protocol in the control group.

In each patient, an 18-gauge intravenous catheter was placed in an antecubital vein of the upper limb, and contrast medium was injected using an automatic double-syringe injector (MedradStellant, Pittsburgh, PA, USA). A fixed dose of contrast medium volume (Iobidtritol 350—Xenetix, Guerbet, Aulnay, France) according to each protocol (60 mL for CCTA and pre-TAVR, 50 mL for CTPA), with a flow rate of 4.5 mL/s for CCTA and pre-TAVR CTA and 3.5 mL/s for CTPA, followed by saline flushing (volume 40 mL for CCTA and 50 mL for CTPA and pre-TAVR protocols) was administered. With a trigger level of 120 HU and an 8 s delay, the bolus-tracking approach (B-T) was used to obtain the start of the scanning. For CCTA and pre-TAVR CTA, the trigger area was manually positioned in the proximal ascending aorta; for CTPA, it was placed in the common pulmonary trunk.

Prior to the CCTA examination and pre-TAVR CTA protocols, metoprolol (5–10 mg) was intravenously given to patients if their baseline heart rate (HR) was greater than 65 beats per minute (bpm) and they did not have a contraindication for β-blockers.

Scan parameters and CM details are summarized in Table 1.

### 2.3. Reconstruction Algorithms

CT images were reconstructed with MBIR (IMR—Philips Healthcare, Cleveland, OH, USA, level 1) and HIR (iDose—Philips Healthcare, level 4) for the study and control group, respectively. IMR level 1 and iDose level 4 were used as standards of reference for clinical practice, according to the vendor’s specification.

### 2.4. Image Analysis

Two radiologists with 10 and 15 years of experience in cardiovascular imaging, blinded to clinical data, randomly evaluated the CT images of both groups, previously anonymized by a radiologist in training.

For the quantitative analysis, the following vascular structures’ lumens were manually marked with a circular region of interest (ROI): the left ventricle; the ascending aorta; the left, right, and circumflex coronary arteries for CCTA; the main, right, and left pulmonary arteries for CTPA; the aortic arch; and the aorta at the renal artery level for pre-TAVR CTA. The vessel contrast enhancement (namely “contrast”) was expressed as the mean attenuation value (Hounsfield unit or HU) in the axial native images. To increase robustness in identifying the above-reported vascular structures and to obtain standardized results, the two radiologists underwent a specific test session before acquiring data.

The ROI sizes were as large as possible depending on the artery caliber, avoiding wall calcifications and atherosclerotic plaque.

Image noise was defined by using the standard deviation (SD) HU of each ROI directly measured using the specific PACS tool (IMPAX 6—AGFA Healthcare, Belgium).

Signal-to-noise ratio (SNR = mean artery attenuation/mean artery standard deviation) and contrast-to-noise ratio (CNR) were computed using the formulas previously reported by Park et al. [21].

A 4-point Likert scale was used to evaluate subjective image quality based on the presence of artifacts, contrast, and spatial resolution. A score of 4 indicated excellent image quality (very low image noise and high sharpness), a score of 3 indicated good image quality (low noise and good sharpness), a score of 2 indicated fair image quality (moderate noise and average sharpness), and a score of 1 indicated poor image quality (high noise and low sharpness). All data regarding qualitative and quantitative image analysis were obtained by consensus between the two readers. In the case of significant differences between the two readers’ evaluations, a third expert opinion by a radiologist with 15 years of experience was considered.

### 2.5. Radiation Dose Quantification and Acquisition Time

The CT dose-length product (DLP, mGy·cm) and the CT dose index (CTDIvol, mGy) were recorded for each scan in order to analyze the radiation dose exposure. Using the established formula ED = k · DLP, where k is the region-specific normalized effective dose (mSv/mGycm) obtained from the publication by Deak et al. [22], we also computed the effective radiation dose (ED) of each CT study. The region-specific conversion coefficients, indifferent for 80 kVp and 100 kVp protocols, were 0.026 mSv/mGycm for CCTA studies, as recently proposed by Trattner et al. [23]; k = 0.0146 mSv/mGycm in CTPA examinations; and the for pre-TAVR CT study, a mean region-specific conversion coefficient k= 0.017 mSv/mGycm was used as a combination among chest, abdominal, and pelvic conversion coefficients, as described by Goetti et al. [24].

For each examination we collected the mean reconstruction time (images/s) directly from the CT scanner.

### 2.6. Statistical Analysis

Categorical variables are expressed as numbers and percentages and were compared with the χ2 test using the Bonferroni correction. All continuous variables were expressed as means. The Shapiro–Wilk test was used to assess normal distribution. The Mann–Whitney U test or Student’s *t*-test were used to evaluate differences between the study and control groups.

All tests were two sided, and a *p*-value < 0.05 was considered to be statistically significant. All statistical analyses were performed with commercially available software (Med Calc 14.8.1, Mariakerke, Belgium).

## 3. Results

### 3.1. Patient Demographics

**Figure 1:**
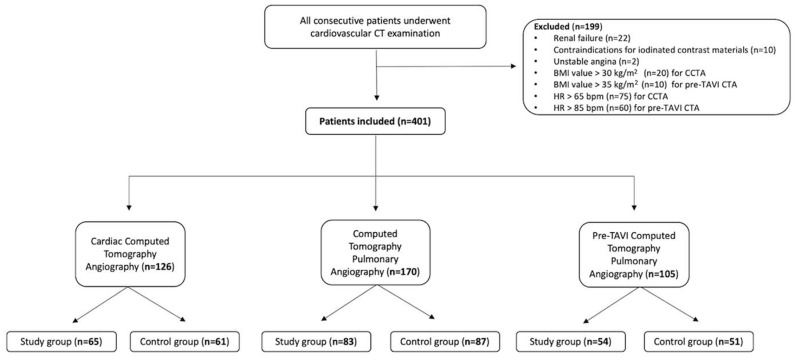
Flow chart showing patients included in our study, who underwent CCTA, CTPA, or pre-TAVR CTA. CTA study group were reconstructed with the MBIR algorithm, while control group with standard HIR.

After excluding 199 patients due to renal failure (n = 22), contraindication for iodinated contrast materials (n = 10), affected by unstable angina (n = 2), BMI higher than 30 (n = 20) and 35 kg/m^2^ (n = 10), and HR higher that 65 and 85 bpm (n = 75 and n = 60, respectively), we enrolled a final cohort of 401 patients, of whom 126 (31.4%) were enrolled for CCTA [n = 65 (51.6%) and n = 61 (48.4%) for the study and control groups, respectively], 170 (42.4%) for CTPA [n = 83 (57.6%) and n = 87 (42.4%) for the study and control groups, respectively], and 105 (26.2%) [n = 54 (51.4%) and n = 51 (48.6%) for the study and control groups, respectively]. The selection process with subgroup division is summarized in the flowchart (Figure 1).

The clinical characteristics of the study and control group patients are summarized in Table 2. No statistically significant differences in terms of sex, age, BMI, baseline HR, and β-blocking between the two groups were found (all *p*-values > 0.05).

### 3.2. Image Analysis Results

#### 3.2.1. CCTA

**Figure 2:**
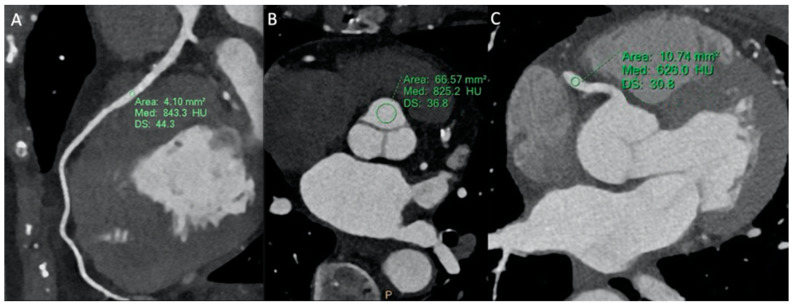
An 80 kVp 256-MDCT combined with the MBIR reconstruction technique. Low-dose coronary angiography study with the following dose values recorded: DLP: 27.1 mGy*m; CTDIvol: 2.1 mGy; and a calculated effective radiation dose (ED) of 0.7 mSv. Images of cardiac CT MBIR study with MPR reconstruction. (**A**) Curved reconstruction of left anterior descendant artery showing myocardial bridging, with good visualization of the artery wall due to high SNR and CNR. The ROI drawn in the intramyocardial tract shows a high attenuation value (843 HU) and low image noise (44 HU). (**B**) Aortic valve MPR reconstruction showing a good attenuation value (825 HU) and a low noise level (37 HU), allowing for an optimal evaluation of aortic sinuses. (**C**) Axial native image showing high attenuation value in the proximal tract of the right coronary artery.

The mean attenuation values (HU) of the proximal tract of the left anterior descending artery (LAD), left circumflex artery (LCx), and right coronary artery (RCA) were significantly higher in the study group than in the control group (619.7 ± 99.3 vs. 475.1 ± 105.2; 626.3 ± 82.5 vs. 434.8 ± 94.3; and 618.5 ± 109.3 vs. 445.7 ± 78.5, *p* < 0.001) (Figure 2). Similar results were found regarding vascular enhancement of the left ventricle (591.9 ± 121.8 vs. 519.7 ± 117.5, *p* = 0.001) and ascending aorta (624.5 ± 117.8 vs. 489.3 ± 101.4, *p* < 0.001).

The noise values were lower for the study group compared to the control group in all anatomic districts analyzed, in particular in coronary arteries (21.1 ± 3.3 vs. 28.4 ± 6.1 for LAD; 22.3 ± 3.1 vs. 30.1 ± 8.3 for LCx; and 21. ± 2.9 vs. 29.3 ± 7.4 for RCA, *p* < 0.001). 

Consequently, CNR and SNR were higher in the study group than in the control group, with a significant statistical difference (Table 3).

The mean image computational time to reconstruct raw image data was significantly higher in the study group compared to the control group (5.1 ± 1.7 vs. 18.3 ± 4.3 images/s, *p* < 0.001).

#### 3.2.2. CTPA

**Figure 3:**
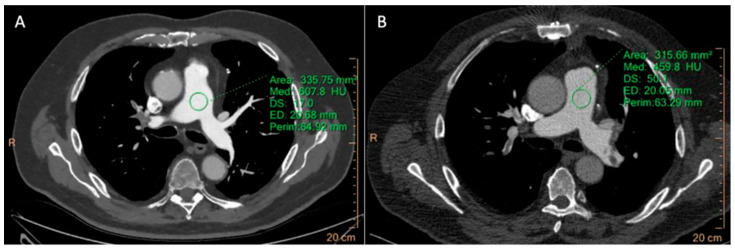
Two 65-year-old (**A**) and 71-year-old (**B**) men both underwent 256-row CT pulmonary angiography study, showing pulmonary embolism. The MBIR algorithm (**A**) shows a higher attenuation value of 608 HU with a lower noise value of 17 HU compared to HIR (**B**) reconstruction (460 ± 50 HU).

Vascular enhancement of the whole pulmonary arterial system was significantly higher in the study group in comparison with the control one, in particular in the main pulmonary trunk (697.9 ± 10.7 vs. 334.8 ± 23.3, *p* < 0.001) and in the right and left pulmonary arteries (651.4 ± 12.1 vs. 318.3 ± 26.8, and 644.6 ± 11.8 vs. 302.8 ± 19.4, *p* < 0.001) (Figure 3).

Image noise was significantly lower in the study group in comparison with the control one in all anatomic districts (15.1 ± 2.5 vs. 19.6 ± 4.3 in the main pulmonary trunk, 17.4 ± 3.3 vs. 20.2 ± 4.1 in the right pulmonary artery, and 17.2 ± 3.0 vs. 20.0 ± 3.9 in the left one, *p* < 0.001).

Accordingly, the CNR and SNR for the main left and right pulmonary arteries were significantly higher for the MBIR algorithm compared with HIR (*p* < 0.001) (Table 3).

The mean MBIR image-reconstruction time was 81.3 ± 8.9 s (8.5 ± 2.8 images/s), while the mean HIR image-reconstruction time was 65.2 ± 7.8 s (11.7 ± 3.2 images/s) (*p* < 0.001).

#### 3.2.3. Pre-TAVR CTA

**Figure 4:**
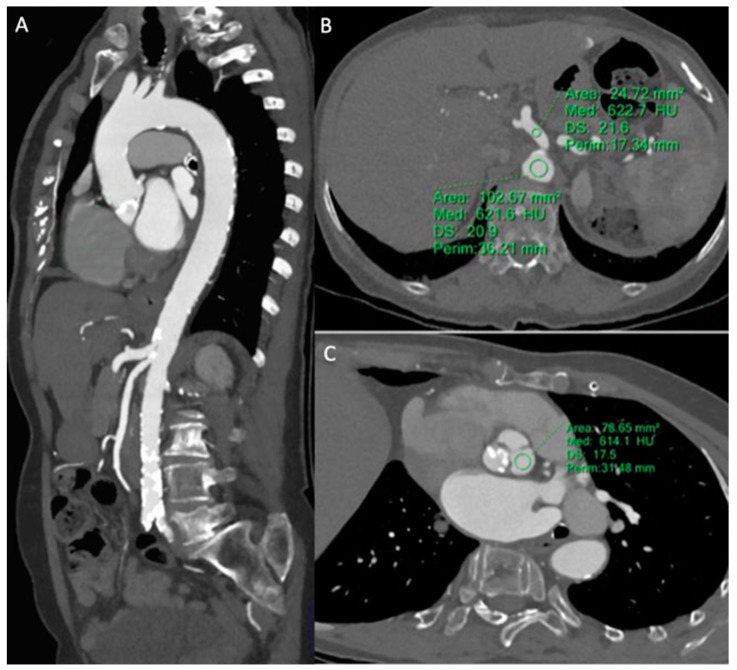
Pre-TAVR CTA with the MBIR algorithm—MIP parasagittal (**A**) reconstruction of the entire aorta. An optimal and homogeneous arterial opacification was obtained at each different aortic level with a low contrast medium volume of 60 mL and an intra-vessel density of up to 622 HU in the abdominal aorta at the celiac trunk level (**B**) with low noise values of 13 and 21 HU, respectively. (**C**) CT reconstruction of the aortic bulb correctly shows calcifications of the non-coronary aortic sinus and a high attenuation value of 614 ± 17 HU.

Aortic enhancement was significantly higher both at the arch and at the emergency of renal arteries in the study group in comparison with the control one (533.6 ± 79.9 vs. 379.6 ± 23.2, and 523.9 ± 82.7 vs. 345.7 ± 31.5, respectively, *p* < 0.001), while the noise was significantly lower (14.2 ± 3.5 vs. 19.2 ± 6.5, and 16.2 ± 3.1 vs. 21.2 ± 7.3, *p* < 0.001) (Figure 4).

Consequently, SNR and CNR were significantly higher in the study group in comparison with the control one (all *p* < 0.001) (Table 3).

The mean image reconstruction time was 201 ± 14.3 s (7.9 ± 2.6 images/s) for the MBIR group and 150.9 ± 12.6 s (10.1 ± 3.6 images/s) for the HIR group (*p* < 0.001).

### 3.3. Image Quality Results

The subjective image quality of CCTA and CTPA study groups was significantly higher than control groups [MBIR: 4 (IQR = 3–4) vs. HIR: 3 (2–4), for both CCTA and CTPA; *p* < 0.01, *p* < 0.001, respectively] (Figure 2 and Figure 3), while overall image quality rating scores were similar between them [4 (3–4) both], without reaching statistical significance (*p* = 0.670).

### 3.4. Radiation Dose Results

Significantly lower CTDIvol was achieved in the study groups compared to the control groups for all protocols (4.32 ± 1.46 vs. 10.33 ± 1.75, 5.92 ± 1.09 vs. 9.82 ± 3.67, and 8.59 ± 3.28 vs. 27.33 ± 5.89, for CCTA, CTPA and pre-TAVR, respectively, all *p* < 0.001), as shown in Table 4.

Similar results were obtained regarding DLP, with a significant reduction in dose exposure for patients enrolled in the study group in comparison with the control one (63.90 ± 32.51 vs. 147.9 ± 33.41, 211.82 ± 31.95 vs. 355.56 ± 3.51, and 588.15 ± 223.87 vs. 1600.1 ± 339.2, for CCTA, CTPA and pre-TAVR, respectively, all *p* < 0.001).

The study group in the CCTA protocol had an average reduction in DLP and ED of 56.9% and 55.8%, respectively, in comparison to the control group, while the estimated dose index (CTDIvol) indicated a reduction of 58.2%. Comparing the CTPA study group to the CTPA control group, we found a 40.4% drop in DLP and ED and a 39.7% decrease in CTDIvol with low-dose CT.

Finally, we found a significant difference between the study group and the control group in terms of DLP and effective dose (ED) in CTA for TAVR planning, with an average reduction of 63.3% and 57.2%, respectively.

## 4. Discussion

Our study confirmed the usefulness of low-kVp CT protocols with fixed contrast media volume, not fixed for patients’ weight, in the evaluation of the most important cardiovascular districts. Particularly, we demonstrated their ability to increase vascular enhancement, expressed as attenuation values; reduce image noise quantitatively, expressed as standard deviation; and consequently increase SNR and CNR in all vascular districts analyzed.

The application of 80 kVp protocols in CCTA combined with the MBIR algorithm helps to increase vascular enhancement in all coronary arteries, as previously demonstrated by Komatsu et al. [25] and Cao et al. [26]. Moreover, we demonstrated that image noise was quantitatively lower in the MBIR images in comparison with the HIR ones, and the SNR and CNR were significantly higher, allowing for a better evaluation of the analyzed vessels. These aspects were partially demonstrated [27] by comparing 120 and 100 kVp protocols, while in our study we demonstrated that similar promising results can be achieved by using 80 kVp in all patients with BMI not higher than 30 kg/m^2^. The reduction in kVp allowed for not only an increase in vascular enhancement due to photoelectric effect, but also a significant reduction in radiation dose exposure (1.66 ± 0.85 vs. 3.75 ± 1.26 mSv in the study and control group, respectively), as demonstrated by Park et al. [21].

We obtained similar results for CTPA, considered nowadays as the reference standard for the evaluation of pulmonary embolism: our data showed a significant increase in vascular enhancement, a decrease in quantitative image noise, and an increase in SNR and CNR in the whole pulmonary arterial system in a low-kVp protocol reconstructed with the MBIR algorithm. In this field, since as early as 2012, Viteri-Ramírez et al. [28] have demonstrated the clinical usefulness of low-dose protocols for the assessment of pulmonary embolism, as confirmed by further studies [29,30,31].

Comparable results were found for patients who underwent CTA for TAVR planning: the use of low-kVp protocols helps increase vascular enhancement, reduce contrast media volume and, if applied to the MBIR algorithm, reduce image noise significantly [32,33,34].

The present study aimed to confirm the above-mentioned previously demonstrated results by prospectively enrolling a large series of patients referred for cardiovascular CT examination, demonstrating the great relevance of low-dose protocols applied in clinical practice. In fact, the use of the 80 kVp protocol combined with mAs modulation, compared with a standard 100 kVp protocol, led to a reduction in the overall radiation dose by 55.8% in CCTA, by 40.4% in CTPA, and up to 63.3% in CTA for TAVR planning.

The obtained results, also in terms of overall image quality, are due to the combination of low-kVp protocols and the MBIR algorithm, as previously demonstrated in a phantom study by Löve et al. [35]: the application of a model-based approach strongly increases attenuation values, reduces image noise, and increases both SNR and CNR in comparison with HIR and FBP. These data were confirmed as useful in clinical practice in the current literature, not only in the cardiovascular imaging but in all anatomic districts, including brain [36,37], lung [38], and abdomen [39,40,41].

The application of low-kVp protocols allows for a reduction in contrast media volume thanks to the higher attenuation of iodine at low kVp, approaching the K-edge of iodine. As is known, in cardiovascular imaging the most important contrast media features to be considered for obtaining diagnostic image quality are the bolus shape and the enhancement peak. Moreover, along with technological CT improvements, scan time is nowadays very low and can mimic the administered bolus, thus leading to avoidance of useless higher-volume protocols for angiographic studies [42]. This approach reduces the risk of acute kidney injuries [43] and other risks induced by contrast media injection, as underlined by the ESC 2019 guidelines [6].

On the other hand, low-KVp CT scans may increase beam hardening artifacts, especially in case of aortic valve calcifications, coronary calcified plaque, or prosthesis [44]. However, MBIR can reduce beam hardening artifacts in comparison with HIR, as previously demonstrated [45,46,47].

One of the greatest limitations of MBIR reported in the literature is the plastic appearance of final images, as reported by Barras et al. [48]. In our series, we found that MBIR images are slightly superior in comparison with HIR through a subjective point-of-view, probably due to the use of very thin slices. The second most important reported limitation of MBIR is the reconstruction time [42]. In our study, we confirmed that MBIR is slower in comparison with HIR; however, this is acceptable for its application in everyday clinical practice.

The present study has the following limitations: first of all, we could not directly compare MBIR-CT and HIR-CT angiographic exams as we investigated two different cohorts of patients to limit the patients’ radiation dose exposure. Patients with BMI > 30 and > 35 kg/m^2^ were not included in the study, and this aspect could have introduced a selection bias. Therefore, hemodynamic differences may have partially affected the study results, although the bolus-tracking technique was used in all patients to match the contrast agent injection as much as possible. Moreover, we could not compare the two different reconstruction algorithms using the same slice thickness, because thinner slices in the HIR group would have certainly increased image noise, making images non-diagnostic and therefore not applicable in everyday clinical practice.

## 5. Conclusions

Our study demonstrated that low-kVp CT protocols combined with the MBIR algorithm can be routinely used in clinical practice, and they allow for the correct performance CT angiographic examinations with high-quality images, using a fixed dose of contrast media, leading to a reduction in radiation dose exposure compared to standard CTA protocols.

## Figures and Tables

**Table 1:** Technical data for study and control groups.

CT Scan Parameters	CCTA		CTPA		Pre-TAVR CTA	
	Study Group	Control Group	Study Group	Control Group	Study Group	Control Group
Tube voltage (kV)	80	100	80	100	80	100
Tube current (mAs)	Automated	Automated	Automated	Automated	Automated	Automated
Gantry rotation time (s)	0.27	0.27	0.27	0.27	0.33	0.33
Slices	256	256	256	256	256	256
Matrix	512 × 512	512 × 512	512 × 512	512 × 512	512 × 512	512 × 512
Pitch	Prospective gating	Prospective gating	0.17	0.17	0.30	0.30
FOV (mm)	250	250	350	350	350	350
Thickness/increment (mm)	0.67/0.34	1/1	0.8/0.4	1/0.5	0.8/0.4	1.0/1.0
CM	Iobiditrol 350	Iobiditrol 350	Iobiditrol 350	Iobiditrol 350	Iobiditrol 350	Iobiditrol 350
CM volume (mL)/flow rate (mL/s)	60/4.5	60/4.5	50/3.5	50/3.5	60/3.5	60/3.5
Saline volume (mL)/flow rate (mL/s)	40/4.5	40/4.5	50/3.5	50/3.5	50/3.5	50/3.5
Reconstruction algorithm	MBIR	HIR	MBIR	HIR	MBIR	HIR

**Table 2:** Patients characteristics.

N = 401	CCTA			CTPA			Pre-TAVR CTA		
	Study Group (n = 65)	Control Group (n = 61)	*p*-Value	Study Group (n = 83)	Control Group (n = 87)	*p*-Value	Study Group (n = 54)	Control Group (n = 51)	*p*-Value
M/F (n)	35/30	33/28	>0.05	42/41	42/45	>0.05	30/24	23/28	>0.05
Age (yo, mean ± SD)	67.4 ± 12.5	62.86 ± 10.46	>0.05	64.45 ±13.44	65.24 ± 12.47	>0.05	72.55 ± 13.44	73.88 ± 13.15	>0.05
BMI (kg/m^2^, mean ± SD)	26.3 ± 3.87	26.8 ± 4.95,	>0.05	23.7 ± 2.1	22.9 ± 1.5	>0.05	24.1 ± 2.6	25.66 ± 4.33	>0.05
Heart rate (bpm, mean ± SD)	62.7 ± 5.29	61.5 ± 8.2	>0.05	-	-		75.3 ± 7.66 (n = 4 β-blocked)	76.2 ± 9.1 (n = 3 β-blocked)	>0.05

**Table 3:** Quantitative image evaluation.

	Arterial Level	Study Group HU	Control Group HU	*p*-Value	Study Group SNR	Control Group SNR	*p*-Value	Study Group CNR	Control Group CNR	*p*-Value
CCTA	LV	591.97 ± 121.8	519.76 ± 117.52	0.001	15.77 ± 3.54	11.14 ± 5.33	<0.001	19.61 ± 6.57	14.65 ± 8.44	<0.001
	Aorta	624.58 ± 117.8	489.33 ± 101.45	<0.0001	16.73 ± 4.11	9.85 ± 7.15	<0.0001	21.11 ± 6.9	13.77 ± 8.39	<0.0001
	LAD-prox	619.77 ± 99.31	475.17 ± 105.21	<0.0001	13.45 ± 6.71	7.79 ± 5.93	<0.0001	20.28 ± 9.6	12.87 ± 10.4	<0.0001
	LCx-prox	626.30 ± 82.54	434.89 ± 94.3	<0.0001	13.84 ± 7.24	7.55 ± 6.24	<0.0001	21.05 ± 7.61	10.85 ± 8.52	<0.0001
	RCA-prox	618.53 ± 109.3	445.7 ± 78.56	<0.0001	14.68 ± 7.25	8.22 ± 6.13	<0.0001	22.36 ± 8.8	11.42 ± 9.19	<0.0001
CTPA	MPA	697.91 ± 10.76	334.87 ± 23.3	<0.0001	62.14 ± 17.3	20.47 ± 13.5	<0.0001	60.7 ± 19.45	10.77 ± 5.76	<0.0001
	LPA	644.61 ± 11.88	302.89 ± 19.45	<0.0001	59.88 ± 18.2	19.5 ± 9.87	<0.0001	57.43 ± 15.2	13.98 ± 6.32	<0.0001
	RPA	651.43 ± 12.17	318.31 ± 26.84	<0.0001	55.16 ± 16.1	22.54 ± 10.7	<0.0001	54.6 ± 13.33	14.6 ± 8.69	<0.0001
Pre-TAVR CTA	Aortic arch	533.60 ± 79.92	379.66 ± 23.28	<0.0001	24.46 ± 7.59	16.23 ± 6.54	<0.0001	27.29 ± 8.70	13.77 ± 4.82	<0.0001
	Aorta at renal a.	523.9 ± 82.7	345.7 ± 31.55	<0.0001	21.30 ± 5.88	13.69 ± 4.23	<0.0001	26.70 ± 7.96	15.02 ± 7.21	<0.0001

**Table 4:** Radiation dose exposure values for study and control groups.

	CCTA		CTPA		Pre-TAVR CTA	
Data/Protocol	Study Group	Control Group	Study Group	Control Group	Study Group	Control Group
CDTIvol (mGy)	4.32 ± 1.46	10.33 ± 1.75	5.92 ± 1.09	9.82 ± 3.67	8.59 ± 3.28	27.33 ± 5.89
DLP (mGy∙cm)	63.90 ± 32.51	147.9 ± 33.41	211.82 ± 31.95	355.56 ± 3.51	588.15 ± 223.87	1600.1 ± 339.2
ED (mSv)	1.66 ± 0.85	3.75 ± 1.26	3.09 ± 0.46	5.19 ± 1.79	10.00 ± 3.81	23.36 ± 4.7

## Data Availability

Dataset available on request from the authors.
